# Single-nucleus multiomic atlas of ALS primary motor cortex nominates neuroprotective WDR49-expressing astrocytes

**DOI:** 10.21203/rs.3.rs-9853460/v1

**Published:** 2026-06-01

**Authors:** Sam Bonsall, Rodrigo Siqueira Kazu, Marianne King, Diana Leung, Farah Y Mahiddine, J Robin Highley, Andrew Strange, Yongzhuo Chen, Matilde Sassani, Sara Cabras, Katie M Bowden, Hollie E Wareing, Mahmoud K Eldahshoury, James R Boyne, Mark Collins, Emma Marie Monte, Calum Harvey, Sarah B Gornall, Akanksha Jangid, Connie Treanor, Tobias Moll, Charlotte H van Dijk, Yiliang Huang, Alex E Urban, Pamela J Shaw, Ryan J H West, Kevin P Kenna, Eran Hornstein, Sai Zhang, Johnathan Cooper-Knock, Jingtian Zhou, Michael P Snyder

**Affiliations:** 1 Sheffield Institute for Translational Neuroscience (SITraN), University of Sheffield, Sheffield, UK; 2 Arc Institute, Palo Alto, CA, USA; 3 Department of Biostatistics, University of Florida, Gainesville, FL, USA; 4 Rita Levi Montalcini’ Department of Neuroscience, University of Turin, Turin, Italy.; 4 Centre for Biomedical Science Research, School of Health, Leeds Beckett University, Leeds, UK; 5 School of Biosciences, University of Sheffield, Sheffield, UK.; 6 Department of Medicine, Stanford University School of Medicine, Stanford, CA, USA; 7 Department of Translational Neuroscience, UMC Utrecht Brain Center, University Medical Center Utrecht, Utrecht, The Netherlands; 8 Departments of Molecular Genetics and Molecular Neuroscience, Weizmann Institute of Science, Rehovot, Israel; 9 Department of Biomedical Informatics & Data Science, Yale University School of Medicine, New Haven, CT, USA; 10 Department of Genetics, Center for Genomics and Personalized Medicine, Stanford University School of Medicine, Stanford, CA, USA; 11 NIHR Sheffield Biomedical Research Centre, Sheffield, UK

## Abstract

Amyotrophic lateral sclerosis (ALS) causes selective neurodegeneration in primary motor cortex, yet cell-type-specific molecular changes driving this vulnerability remain poorly understood. We present an integrated single-nucleus RNA- and ATAC-sequencing atlas of 778,330 nuclei from the primary motor cortex of 140 genetically characterised donors. ALS is associated with widespread transcriptional reprogramming driven by a common set of transcription factors (TFs) across multiple cell-types. Astrocytes harbour the most differentially expressed genes. Within astrocytes, a WDR49-expressing subpopulation is spatially associated with TDP-43 pathology, and genetic variants within *WDR49* confer risk for both sporadic and monogenic autosomal dominant ALS. In patient-derived induced astrocytes, WDR49 protein abundance predicts the survival of co-cultured neurons. WDR49 localises to PML nuclear bodies, where it regulates astrocyte reactivity and secretion of EVs containing protein chaperones. Together, these *in vivo* and *in vitro* findings suggest that WDR49+ astrocytes mount a compensatory secretory response to extracellular protein aggregates, and that loss of this capacity lowers the threshold for ALS pathogenesis.

## Introduction

Amyotrophic lateral sclerosis (ALS) is a fatal neurodegenerative disease characterised by progressive and selective degeneration of upper motor neurons in the primary motor cortex and of lower motor neurons in the spinal cord.^5^ The neuropathological hallmark of more than 98% of cases is cytoplasmic mislocalisation and nuclear depletion of TDP-43, which accumulates in ubiquitinated inclusions within neurons and glia.^6^ The burden of TDP-43 pathology at postmortem predicts the degree of neuronal loss,^7^ and postmortem staging of TDP-43 spread suggests that pathology propagates through anatomically connected circuits in a sequential manner consistent with prion-like seeding.^8,9^ Ten percent of ALS is autosomal dominant, and in the remaining 90%, the estimated heritability is approximately 50%.^10^ The most frequently identified genetic risk factor is a G4C2-repeat expansion of C9orf72,^11^ which is probably not pathogenic in isolation^12^ and is often accompanied by an additional genetic driver.^13^ Despite this high heritability, the upstream cellular events that initiate and propagate neurodegeneration remain incompletely understood, and the majority of patients lack an identified causal genetic variant.^14^

Converging lines of evidence implicate astrocytes as upstream contributors to neurodegeneration in ALS. Astrocytes derived from ALS patient postmortem tissue are toxic to neurons in culture,^14^ and patient-derived induced astrocytes recapitulate this neurotoxic phenotype irrespective of genetic background.^15^ Consistent with an upstream role, rare high-impact ALS-associated variants are enriched within astrocyte-accessible chromatin relative to other CNS cell types, and transcriptomic studies have identified extensive gene expression changes in ALS astrocytes that correlate with inflammatory and reactive programmes.^3,16^ However, astrocytes are not a homogeneous population; single-nucleus studies have defined multiple functionally distinct subtypes in the human brain that differ in their spatial distribution, molecular identity, and relationship to disease.^17,18^ Reactive astrocyte states can be both neurotoxic and neuroprotective depending on the inflammatory context,^19,20^ yet the molecular identity of disease-relevant astrocyte subpopulations in ALS, and in particular whether a neuroprotective subtype exists whose failure could contribute to disease progression, has not been established. Critically, despite the strong genetic enrichment within astrocyte chromatin, no astrocyte-specific genetic driver of ALS has previously been identified.^21^

Single-nucleus transcriptomic studies have begun to characterise the cellular landscape of ALS patient primary motor cortex, identifying disease-associated transcriptional changes across glial and neuronal populations.^3,16^ These studies have established that non-neuronal cells, particularly astrocytes and microglia, undergo the most extensive transcriptional reprogramming in ALS, and have identified cell-type-specific gene expression signatures that distinguish ALS subtypes. However, transcriptomic data alone cannot resolve the upstream regulatory logic driving these expression changes, as it does not capture the chromatin accessibility landscape that determines which transcription factors and cis-regulatory elements are active in each cell type. Integrated single-nucleus RNA-sequencing and ATAC-sequencing (multiome) addresses this limitation by simultaneously profiling gene expression and chromatin accessibility in the same nucleus, enabling reconstruction of gene regulatory networks (GRNs) that link transcription factor binding at specific enhancers to downstream target gene expression. This approach can distinguish the cell-type-specific regulatory drivers of transcriptional responses.^22,23^ However, no paired multiome atlas of the ALS motor cortex at the scale required to power cell-type-resolved GRN analysis has previously been reported.

We have performed multiome sequencing of primary motor cortex tissue from 70 donors: 25 non-neurological controls, 26 sporadic ALS patients, and 19 patients who suffered ALS associated with G4C2-repeat expansion of *C9orf72*.^11^ We integrated this with another large single-nuclei RNA-sequencing study of motor cortex from ALS patients and controls^3^ to derive a total dataset of 778,330 nuclei including 82,005 astrocyte nuclei. We provide genetic evidence that loss of function of WDR49 is associated with increased risk for both monogenic autosomal dominant ALS^24^ and sporadic ALS.^21^ We characterised astrocytes which express WDR49 (WDR49+) *in vivo* and *in vitro*. WDR49+ astrocytes are neuroprotective and reduced expression of WDR49 is associated with disease severity and neurotoxicity in co-culture. Gene-expression changes in WDR49+ astrocytes are enriched with genetic drivers of ALS including FLOT2, an important protein for EV biogenesis^25^ and a canonical EV marker.^26^ Patient-derived astrocytes reveal that WDR49 is localised to PML nuclear bodies, and loss of WDR49 is associated with impaired astrocyte reactivity including reduced EV secretion of extracellular protein chaperones which could ameliorate propagation of TDP-43 pathology.

## Results

### Single-cell multi-omic atlas of ALS cohort

**Extended Data Figure 1:**
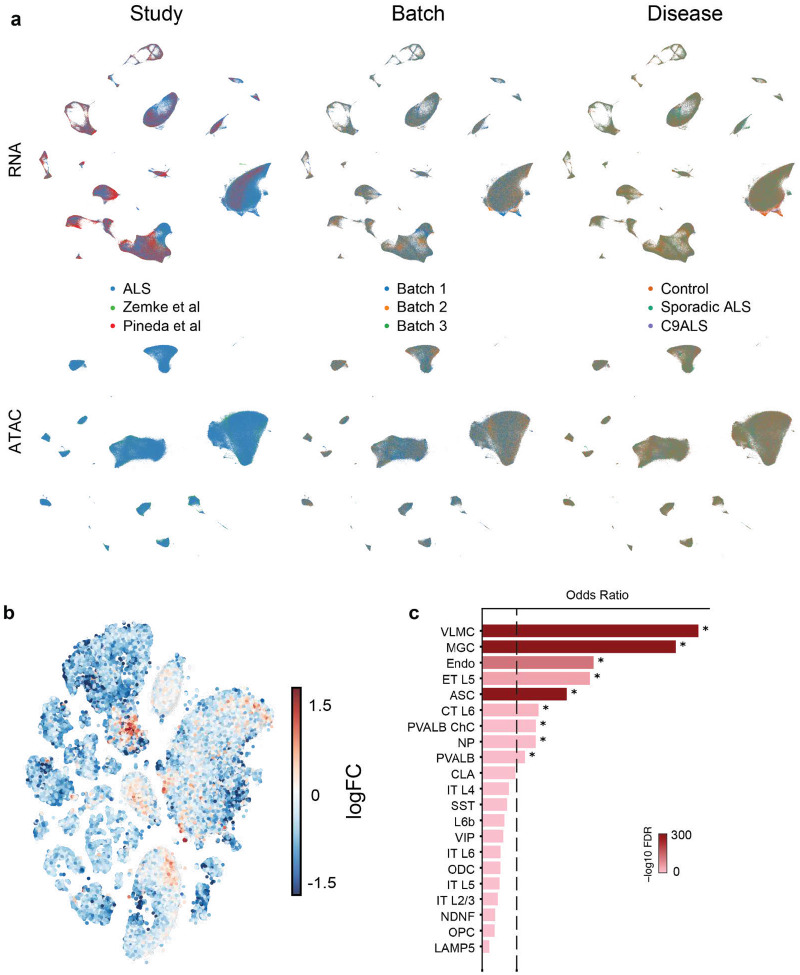
related to ‘Figure 1: Atlas of cell-specific gene regulation changes in ALS primary motor cortex reveals cell-specific genetic drivers of disease’. (**a**) Two-dimensional tSNE embedding of 778,330 single nuclei coloured by cell type annotation coloured by study, batch, and donor disease status. (**b**) ALS-associated changes in cell composition quantified using MILO.^4^ (**c**) Enrichment of ALS-associated genetic risk within cell-specific gene expression. Gene expression was measured in controls from this study; enrichment was measured using scDRS.^2^

**Figure 1 |:**
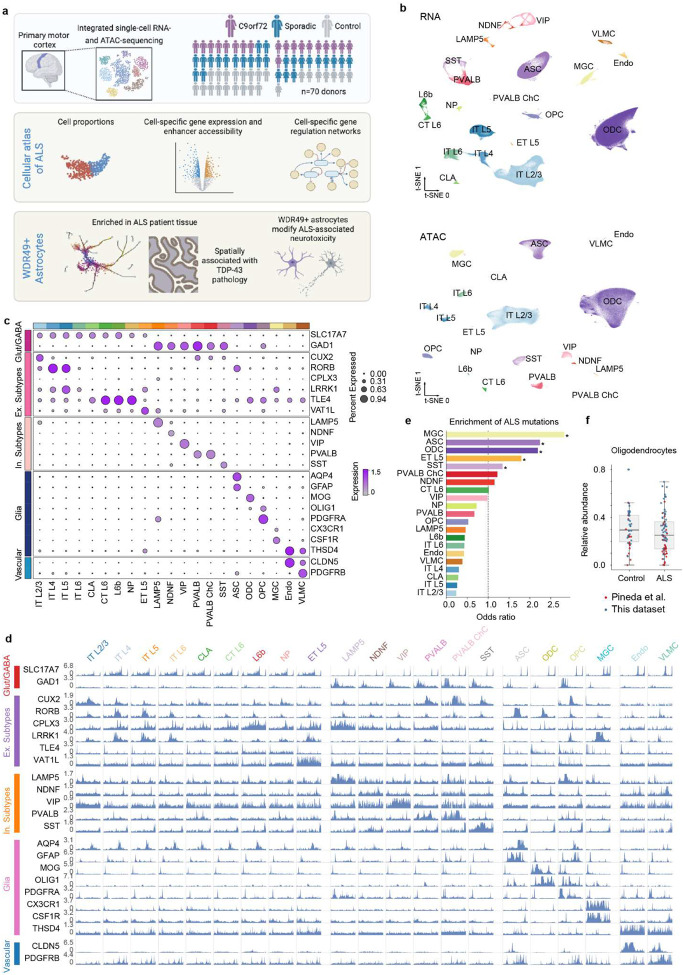
Atlas of cell-specific gene regulation changes in ALS primary motor cortex reveals cell-specific genetic drivers of disease. (**a**) Study overview. We assembled a cohort of n = 70 donors, including C9orf72-ALS and sporadic ALS patients, and age- and sex-matched non-ALS controls, and performed integrated single-nucleus RNA-sequencing and ATAC-sequencing on primary motor cortex post-mortem tissue (top). We characterised cell-specific changes in gene expression and regulation (middle). We identified WDR49+ astrocytes as associated with disease and protective against neuronal toxicity; in vitro modelling revealed that WDR49 resides within PML bodies and moderates astrocyte release of neuroprotective extracellular vesicles (bottom). (**b**) Two-dimensional tSNE embedding of single nuclei coloured by cell type annotation for gene expression (n=778,330, top) and chromatin peak accessibility (n=522,472, bottom). Cell identities were assigned by label transfer from a high-quality reference atlas.^1^ Twenty-two distinct cell populations were identified, spanning excitatory and inhibitory neuron subtypes, glia and vascular cell types. (**c**) Dot plot of canonical marker gene expression from RNA-sequencing across all annotated cell types. Dot size represents the fraction of expressing cells; colour intensity represents the mean expression level. (**d**) Genomic tracks displaying aggregated ATAC-sequencing signal representing chromatin accessibility at canonical marker loci across annotated cell types. (**e**) Enrichment of ALS-associated genetic risk within cell-specific gene expression. Gene expression was measured in a reference dataset;^1^ enrichment was measured using scDRS.^2^ (**f**) Relative abundance of oligodendrocytes per donor in control and ALS groups, shown for two independent cohorts (this study, blue; Pineda et al.,^3^ red). Abbreviations: ASC, astrocytes; ASC WDR49, WDR49-positive astrocytes; ODC, oligodendrocytes; OPC, oligodendrocyte precursor cells; MGC, microglia; IT, intratelencephalic neurons (L2/3, L4, L5, L6); ET L5, extratelencephalic layer 5 neurons; CT L6, corticothalamic layer 6 neurons; L6b, layer 6b neurons; NP, near-projecting neurons; LAMP5, NDNF, VIP, SST, PVALB, PVALB ChC, inhibitory interneuron subtypes; CLA, claustrum neurons; Endo, endothelial cells; VLMC, vascular leptomeningeal cells.

We generated paired single-nucleus transcriptomes and chromatin accessibility profiles from postmortem primary motor cortex of 70 individuals: 25 non-neurological controls, 26 sporadic ALS patients, and 19 patients who suffered ALS associated with G4C2-repeat expansion of *C9orf72* (C9ALS)^11^ (Figure 1a, **Supplementary Table 1**). After quality control and integration with a motor cortex reference atlas,^1^ we annotated 21 cell types comprising 9 excitatory neuronal, 5 inhibitory neuronal, and 7 non-neuronal populations, totaling 586,219 nuclei for RNA and 522,472 for ATAC (Figure 1b). Cell type identity was validated by canonical marker expression and locus-specific chromatin accessibility (Figure 1c,d). Co-embedding with an independent ALS cohort^3^ consisting of 265,079 nuclei to make a total dataset of 778,330 nuclei, confirmed a reproducible cell-type architecture across studies, donors, sexes, and disease status (Extended Data Figure 1a).

We mapped cell-type specific gene expression onto an ALS genetic dataset^21^ to identify which cells are upstream drivers of disease using scDRS.^2^ Rare high-impact ALS-associated variants were enriched in accessible chromatin of microglia (OR = 2.82), astrocytes (OR = 2.24), and oligodendrocytes (OR = 2.19), with more modest enrichment in ET L5 corticospinal neurons (OR = 1.80) and SST interneurons (OR = 1.35) (Figure 1e). Cell-type specific enrichment with genetic risk for ALS is consistent using the reference motor cortex dataset^1^ or control data from this study (Extended Data Figure 1c). The concentration of genetic risk for ALS within astrocyte gene expression is consistent with the idea that astrocytes are upstream drivers of ALS pathogenesis.

We determined compositional changes in cell type proportions between ALS and control donors. Using scCODA^27^, the only significant change in any cell-type between controls and either ALS subgroup was a modest reduction in the proportion of oligodendrocytes in ALS patients (FDR < 0.2), with a decrease in mean proportion from 31.4% to 27.4% (inclusion probability = 0.92, log-effect β = −0.14, Figure 1f, **Supplementary Table 2**). We applied MILO^4^ to measure changes in ‘neighbourhoods’ defined by gene expression signatures, but there was no significant ALS-associated change in the proportion of any cellular ‘neighbourhood’ (Extended Data Figure 1b). Analysis of the correlation between cell proportions, ‘neighbourhood’ proportions and patient survival did not reveal any significant correlations after FDR correction. Together, these suggest that ALS is not associated with large-scale changes in cellular abundance within primary motor cortex.

### Gene expression changes in ALS

**Figure 2:**
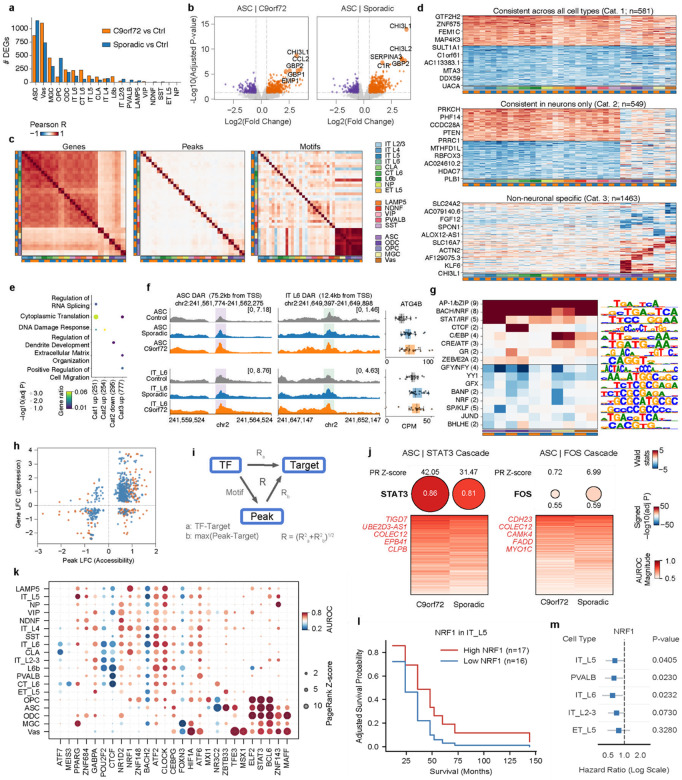
Cell-type-specific transcriptional alterations in ALS motor cortex. **(a)** The number of ALS-associated differentially expressed genes (DEGs) per cell-type. (**b**) Volcano plots of DEGs identified in astrocytes between C9ALS ALS (left panel) or sporadic ALS (right panel) patients compared to controls. (**c**) Heatmaps depicting Pearson correlation between cell types and disease types (row and column colours) for changes in gene expression (left panel), chromatin peak accessibility (middle panel) and TF motif enrichment (right panel). (**d,e**) Heatmap depicting gene expression changes (**d**) or GO enrichment (**e**) of shared and neuronal/glial-specific ALS-associated DEGs. Dot sizes and colours in (**e**) represent −log10 adjusted P-values and proportion of genes belonging to each GO term. Dots with adjusted P-values > 0.1 are not shown. (**f**) ATAC signal of ATG4B flanking peaks (left) and gene expression of ATG4B (right) in astrocytes and intratelencephalic neurons from L6. (**g**) Motif enrichment in top differentially accessible regions (DARs) between ALS patients and controls across disease types and non-neuronal cell types (column colours). (**h**) Comparison of log fold change (LFC) for DEGs and associated DARs. (**i**) Illustration of GRN construction. (**j**) PageRank Z-score (size, text above circles) and AUROC (colour, text in or below circles) of STAT3 and FOS (top) and AUROC of STAT3/FOS target gene expression in astrocytes (bottom) for distinguishing ALS patients and controls. (**k**) PageRank Z-score (size) and AUROC (colour) of ALS-associated TFs across cell types. (**l**) Kaplan-Meier curve depicting survival probability for ALS patients divided by NRF1 expression in intratelencephalic neurons from L5. (**m**) Hazard ratios and p-values for Cox regression analysis of NRF1 regulatory activity (via PageRank score) as a determinant of ALS patient survival.

We determined cell-specific gene expression changes associated with ALS via DESeq2.^28^ Non-neuronal cells, including astrocytes, vascular cells, microglia, and oligodendrocyte-lineage cells, harbored the majority of differentially expressed genes (DEGs), with astrocytes alone accounting for >2,000 DEGs (Figure 2a, **Supplementary Table 3**). Chitinases were up-regulated in astrocytes (Figure 2b) which is consistent with previous literature.^29^ Notably, *C9orf72* is downregulated in C9ALS donors in the combined dataset but only in oligodendrocytes (DESeq2, Log2FC = −0.49, FDR = 6.3e-4).

Next, we examined whether ALS-associated gene expression changes are shared across cell types and disease subgroups. Per gene effect sizes for C9ALS and sporadic ALS are correlated across all cell types (Pearson r = 0.45, Figure 2c, **left panel**). This demonstrates that both ALS subtypes engage similar transcriptional programs despite genetic heterogeneity. Correlations between cell types are weaker than those between ALS subtypes (Figure 2c, **left panel**), but it is notable that ALS-associated gene expression changes within neuronal cell types are more correlated than between neuronal and non-neuronal cell types, or between non-neuronal cell types, indicating shared a shared molecular signature of neuronal pathology.

Inspired by the observed pattern of correlation in gene expression across cell types, we classified the DEGs into three categories: Genes altered consistently across all cell types (Cat. 1; 581 genes), specifically in neurons (Cat. 2; 549 genes), or specific to non-neuronal cells (Cat. 3; 1,463 genes) (Figure 2d). Gene ontology (GO) analysis revealed that the upregulated portion of Cat. 1 genes was enriched for RNA splicing and translational machinery (Figure 2e, **Supplementary table 5**). Upregulated neuronal genes (Cat. 2) were enriched for DNA damage response pathways, consistent with previous reports.^30^ Downregulated genes were enriched for axonal functions including dendrite development and neurotransmitter receptor trafficking, which is consistent with our previous work highlighting distal axon dysfunction to be an upstream driver of ALS.^22^ Cat. 3 genes include upregulated genes associated with cell migration which is consistent with reactive astrogliosis and microglial activation.

### Chromatin changes in ALS

**Extended Data Figure 2:**
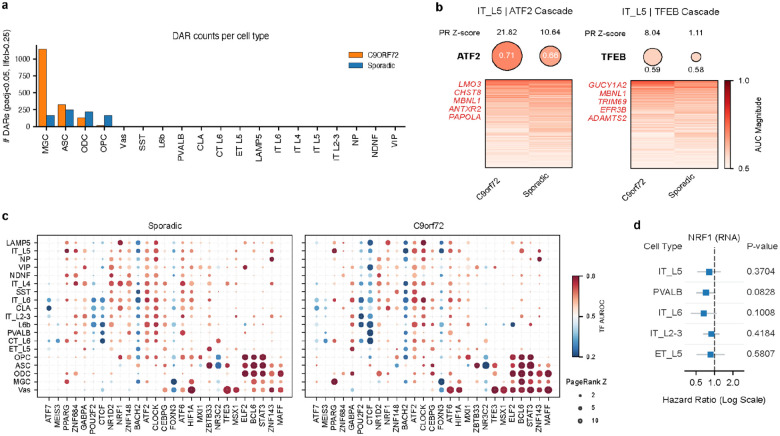
related to ‘Figure 2: Cell-type-specific transcriptional alterations in ALS motor cortex’. (**a**) The number of ALS-associated differentially accessible regions (DAR) per cell-type. (**b**) ageRank Z-score (size, text above circles) and AUROC (colour, text in or below circles) of ATF2 and TFEB (top) and AUROC of ATF2/TFEB target gene expression in intratelencephalic L5 neurons (bottom) for distinguishing ALS patients and controls. (**c**) PageRank Z-score (size) and AUROC (colour) of ALS-associated TFs across cell types, by ALS subtype. (**d**) Hazard ratios and p-values for Cox regression analysis of NRF1 expression as a determinant of ALS patient survival.

In contrast to the broadly shared transcriptional response, observed chromatin accessibility changes associated with ALS were markedly cell-type-restricted. Correlations between cell types were near zero (Pearson r = 0.016; Figure 2c, **middle panel**), indicating that the chromatin landscape within each cell type responds independently to disease. Statistically significant differentially accessible regions (DARs) were found almost exclusively in non-neuronal cells (Extended Data Figure 2a).

Lack of shared DARs between cell-types is in contrast to ALS-associated DEGs. This dissociation between shared transcriptional programs and divergent chromatin changes implies that the same gene can be regulated through different cell-type-specific cis-regulatory elements (CREs). As an example, *ATG4B*, an autophagy gene which is mis-spliced in ALS^31^, is upregulated in both astrocytes and IT L6 neurons (Figure 2f). There is an astrocyte-specific DAR located 75 kb distant from the *ATG4B* transcription start site (TSS) and an independent IT L6-specific DAR located 12 kb distant from the TSS. Both peaks show increased accessibility in ALS consistent with increased *ATG4B* expression, but neither peak is accessible in both cell types.

CREs are usually bound by transcription factors (TFs) to regulate gene expression. We hypothesised that the disparity between ALS-associated DEGs and DARs across cell types may reflect a common set of TFs acting via distinct enhancers. To test this we examined enrichment of TF binding-motifs within disease-associated DARs.^32^ In non-neuronal cells motif enrichment revealed a convergence of inflammation associated TF programs (Figure 2c,g). The AP-1/bZIP family, including FOS, JUN, FRA1/2, ATF3, and BATF, was highly enriched in DARs which were gained in ALS patient samples (Figure 2g), consistent with AP-1’s established role as a master regulator of inflammatory gene expression in activated glia.^33,34^ The BACH/MAF/NRF2 antioxidant response pathway and STAT/IRF cytokine signaling were also enriched in ALS-associated DARs, suggesting coordinated activation of stress-response and inflammatory cascades. Conversely, DARs lost in ALS were depleted of homeostatic regulators, including RONIN/GFY/NFY and YY1, indicating active remodeling of the glial chromatin landscape away from quiescent programs.

### Gene-regulatory network (GRN) changes associated with ALS survival

We have shown that disease-associated gene expression changes which occur in multiple cell-types, are driven through distinct cis-regulatory elements in each cell type. This has implications for our understanding of ALS-associated molecular changes and how they could be reversed in a cell-specific manner. To test our model directly, we linked significantly differentially accessible peaks to differentially expressed genes within 100kb and observed that chromatin and transcriptional changes are broadly concordant in direction (Figure 2h). While this shows that the cis-regulatory changes identified in our chromatin analysis are functionally coupled to downstream gene expression, the relationship between peak and gene effect sizes is noisy, suggesting that chromatin accessibility alone provides an incomplete picture of transcriptional regulation in disease. To identify a higher-order regulatory logic coordinating ALS-associated changes, we constructed cell-type-specific gene-regulatory networks (GRNs) integrating peak-gene correlations, motif annotations, and TF-gene correlation (Figure 2i). A GRN links TFs with downstream target genes through its binding-motifs in CREs whose accessibility is correlated with target gene expression. We applied a PageRank analysis to discover signature TFs,^35,36^ where a disease-association score is propagated through the GRN. The score of a TF increases if the TF expression is a good discriminator of ALS patients and controls, *and* if the TF is predicted to regulate a larger proportion of genes whose expression distinguishes ALS patients and controls (Methods, Figure 2i). As an example, in astrocytes, *STAT3* emerged as a dominant regulatory hub with a high PageRank score in both C9ALS and sporadic ALS, driving a coherent downstream program of gene expression (Figure 2j, **left panel**). In contrast, *FOS* expression is similar in C9ALS and sporadic ALS but its downstream genes discriminate only sporadic ALS, which leads to a higher PageRank score in sporadic ALS (Figure 2j, **right panel**). A similar example is provided in intratelencephalic layer 5 neurons (Extended Data Figure 2b). Together, this suggests that inflammatory chromatin remodeling in ALS astrocytes is orchestrated by multiple convergent TF programs rather than a single master switch.

We identified TFs with the strongest ALS-associated regulatory activity measured by PageRank score, across both ALS subtypes and all cell types (Figure 2k), many of which correspond to disease-associated chromatin accessibility changes at TF-binding motifs (Figure 2g). Hierarchical clustering of these TFs reveal a distinct pattern in neuronal compared to non-neuronal cell types (Figure 2k); subtype-stratified analysis largely recapitulates this structure, supporting the use of a pooled ALS comparison for characterizing shared disease-associated regulatory programs (Extended Data Figure 2c). The neuronal cluster is characterized by TFs associated with homeostasis including *NRF1*, a master regulator of mitochondrial biogenesis and oxidative phosphorylation; and *ATF2*, regulator of apoptotic transcriptional programs. Both *NRF1*^37^ and *ATF2*^38^ have been previously associated with neurodegeneration. Meanwhile, the glial cluster is anchored by *STAT3*, a well-established driver of astrocyte reactivity.^39^

Finally, we asked whether any of the identified TF programs carried prognostic significance for ALS patients. We did not identify any single gene expression change associated with ALS survival after multiple testing correction (**Supplementary Table 4**). However, network-level TF activity, summarized by PageRank scores, captures survival-associated regulatory variation not detectable at the single-gene level (Extended Data Figure 2d). Cox regression of TF PageRank scores against patient survival identified *NRF1* as positively associated with survival in multiple subclasses of excitatory neurons (IT L5: P = 0.04; PVALB: P = 0.02; IT L6: P = 0.02; Figure 2l,m), although did not survive correction for multiple comparisons in any single cell type. Additionally, *NRF1* is a top-ranking regulator of ALS-associated gene expression in our GRN analysis (Fig. 2k). NRF1 is a driver of mitochondrial biogenesis and therefore this result is consistent with our previous work linking neuronal bioenergetic function to ALS survival.^40^

### Failure of WDR49-positive astrocytes is causally related to ALS

**Figure 3:**
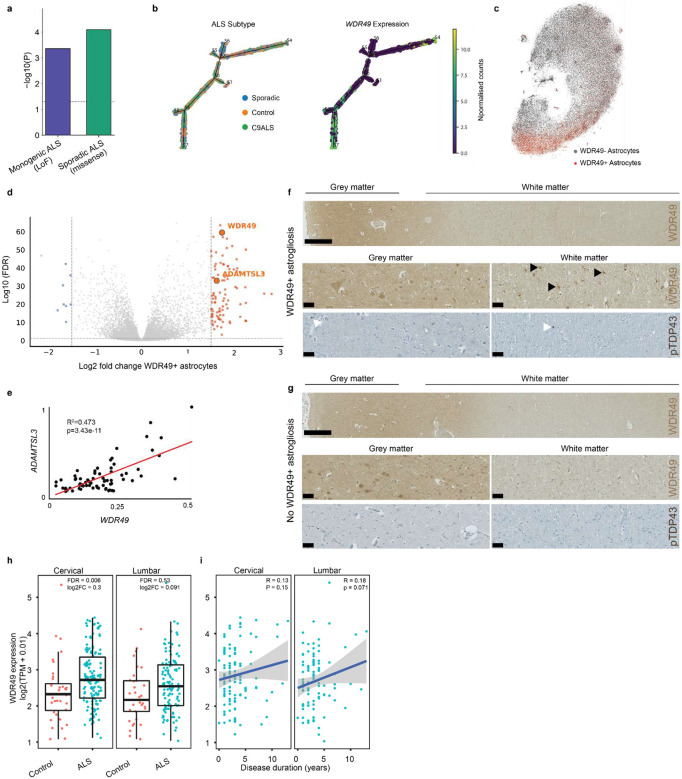
Neuroprotective astrocytes expressing WDR49 (WDR49+) are enriched in ALS patient tissue. (**a**) Rare variant gene burden testing to evaluate enrichment of ALS-associated mutations within WDR49; dotted line p=0.05. LoF = nonsense mutations designated loss-of-function. (**b**) Pseudotime trajectory analysis highlights a branch of WDR49+ astrocytes (S7). (**c**) t-SNE embedding of astrocyte RNA expression illustrating WDR49+ and WDR49− astrocytes. (**d**) Differential gene expression comparing the WDR49+ trajectory branch (S7) to other astrocytes. (**e**) Spearman correlation for WDR49 and ADAMTSL3 expression in pseudobulked astrocyte expression data. (**f-g**) Relationship between WDR49 astrocytosis and TDP43 pathology. Low power WDR49 immunohistochemistry (upper section of both **f** and **g**) with full thickness cortex and underlying white matter in region with (**f**) and without (**g**) WDR49+ astrocytosis (scale bar = 500mm). High power WDR49 (middle section of both **f** and **g**, scale bar = 50mm) and TDP43 (lower section of both **f** and **g**, scale bar = 50mm) immunohistochemistry. Phosphorylated TDP43 inclusions (white arrowhead) are seen in proximity to WDR49+ astrocytes (black arrowheads) including in the overlying cortical grey matter. (**h**) Bulk RNA-Seq from post-mortem cervical and lumbar spine shows a trend to increased WDR49 expression in ALS vs control which is significant in cervical spine (FDR = 0.006). (**i**) WDR49 expression in both cervical and lumbar spine positively correlates with ALS patient survival.

The concentration of ALS genetic risk within astrocyte chromatin (Figure 1e, Extended Data Figure 1c), combined with the predominance of ALS-associated transcriptional changes in this lineage, raises the possibility an astrocyte subpopulation is an upstream driver of disease. WDR49-positive (WDR49+) astrocytes have been described in frontotemporal dementia and Alzheimer’s, and linked to TDP-43 pathology,^41^ but their relevance to ALS has not been examined and the function of WDR49 is not understood. Genetic analyses show that WDR49 mutations increase risk for ALS: rare-variant burden testing in a familial (majority autosomal dominant) ALS cohort identified enrichment of loss-of-function variants within WDR49 (ALS frequency = 0.5%, OR = 15.8, p = 4.4e-4),^24^ but did not connect this association to astrocyte biology. In a broader population of largely sporadic ALS patients we observed a significant enrichment of missense variants in WDR49 (ALS frequency = 2%, OR = 1.3, p = 8.1e-5, SKAT, Figure 3a).^21,42^ The convergence of large disease-associated changes in our astrocyte population, prior literature on the role of WDR49+ astrocytes in neurodegenerative disease, and consistent genetic signal of WDR49 in across familial and sporadic ALS motivated us to characterise WDR49+ astrocytes in patient tissue.

**Extended Data Figure 3:**
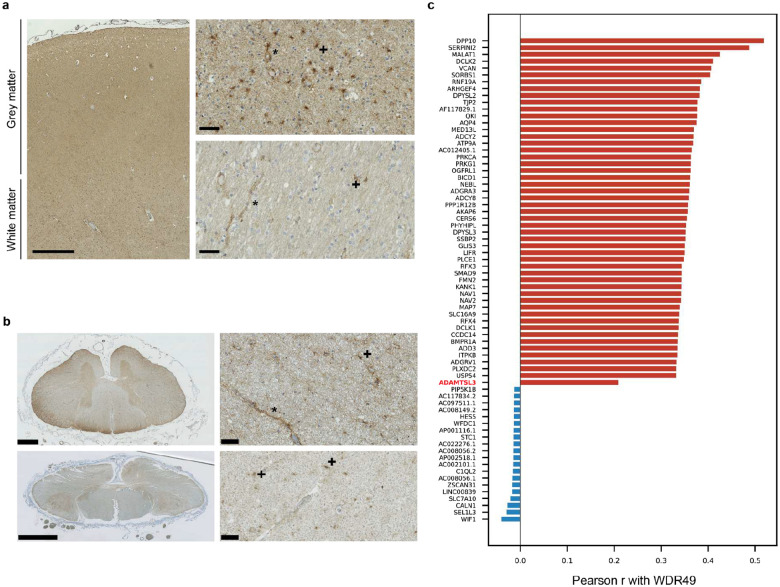
related to ‘Figure 3: Neuroprotective WDR49-positive (WDR49+) astrocytes are enriched in ALS patient tissue.’ (**a**) Immunohistochemistry for WDR49 in ALS (left panel and top panel) and control (bottom panel) motor cortex. Scale bar: left panel, 1mm; right panels, 50μm (**b**) Immunohistochemistry for WDR49 in ALS (bottom panels) and control (top panels) in spinal cord. l cord. Scale bar: left upper panel, 1mm; left lower panel, 2.5mm; right panels, 50μm. +, WDR49+ astrocytes distant from blood vessels; * perivascular WDR49. (**c**) Gene expression correlated (Pearson correlation, FDR<0.05) with WDR49 in WDR49+ astrocytes.

We first asked whether WDR49+ astrocytes are identifiable within our single-nucleus dataset. Trajectory analysis^43^ of the 82,005 astrocytes in our cohort - chosen over discrete clustering because astrocytes occupy a continuum of functional states^44,45^ - recovered a distinct ‘branch’ defined by high *WDR49* expression (Figure 3b,c). Within this branch, *ADAMTSL3* was the most significantly co-expressed gene with WDR49 (Figure 3d,e; Pearson r = 0.74, p = 5.8e-22, Extended Data Figure 3c), which is notable because *ADAMTSL3*+ astrocytes are designated neuroprotective in Alzheimer’s disease.^46^ This is consistent with our genetic analysis showing that loss of function of WDR49 is deleterious and therefore suggesting that the function of WDR49 is neuroprotective. Gene set enrichment analysis of WDR49+ astrocyte-specific expression changes revealed robust upregulation of cytokine-mediated signalling (NES = 2.30–2.31, FDR ≤ 0.001 in both C9ALS and sporadic ALS), TNF signalling (NES = 2.16–2.27, FDR ≤ 0.01), and membrane raft organisation (NES = 2.00–2.14, FDR ≤ 0.01), identifying this population as transcriptionally reactive and a potential mediator of neuroprotective inflammation.

### WDR49+ astrocytes are spatially related to TDP-43 pathology

To study WDR49+ astrocytes histologically in independent tissue, we performed blinded immunohistochemistry on postmortem precentral gyrus and spinal cord corticospinal tract from ALS patients and controls. WDR49+ astrocytes were present in 1 of 4 neurologically healthy controls but detected in 10 of 12 ALS cases (9 sporadic ALS, 3 C9orf72-ALS; Chi-squared, df = 1, p = 0.03, Figure 3f, Extended Data Figure 3a), with a consistent pattern in spinal cord (6 of 7 sporadic ALS cases, 0 of 3 controls, df = 1, p = 0.02, Extended Data Figure 3b). Rather than being uniformly distributed, WDR49+ astrocytes formed discrete foci concentrated in white matter. We wondered whether foci of WDR49+ astrocytes are spatially associated with TDP-43 pathology, which is the hallmark of ALS.^6^ To test this hypothesis we defined 1 mm diameter regions of interest (ROIs) and performed blinded quantification of TDP-43 inclusions in neurons and glia within ROIs containing or lacking WDR49+ astrocytes. ROIs harbouring WDR49+ astrocytes had significantly greater TDP-43 pathological burden in neurons in the overlying cortex (n = 16 ROIs, Wilcoxon signed-rank test, p = 0.015; Figure 3a), glia in the overlying cortex (n = 16 ROIs, p = 0.002), and glia in the same white matter region as the WDR49 astrocytosis (n = 16 ROIs, p = 0.0049), establishing a tight spatial relationship between WDR49+ astrocyte accumulation and TDP-43 pathology. Our genetic evidence suggest that the function of WDR49 is neuroprotective and therefore were surmise that WDR49+ astrocytes may perform a targeted amelioration of TDP-43 pathology.

As a further validation we examined *WDR49* expression in bulk RNA-sequencing data from 154 ALS patient and 49 control postmortem cervical and lumbar spinal cord samples.^47^ *WDR49* expression was significantly elevated in ALS tissue (limma-voom, log_2_FC = 0.3, FDR = 6e-3; Figure 3g). Within the ALS group, *WDR49* expression is positively correlated with patient survival (Pearson r = 0.13; Figure 3h), consistent with a neuroprotective effect.

### WDR49 expression enables astrocytes to manifest a neuroprotective molecular program

**Extended Data Figure 4:**
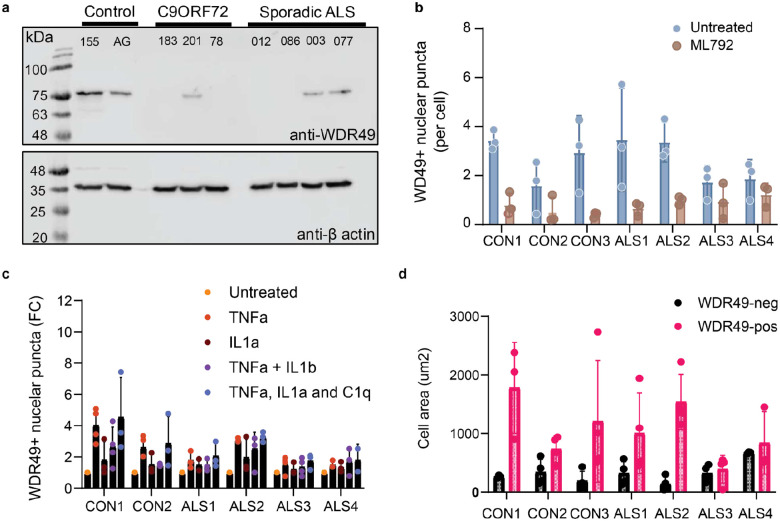
related to ‘Figure 4: WDR49 expression within iAstrocytes (iA) is associated with neurotoxicity.’ (**a**) Immunoblotting for WDR49 across different iA lines, with β-actin for normalization. (**b-c**) Immunocytochemistry quantification of average number of PML bodies and WDR49+ nuclear puncta per iA in individual iA lines after treatment after (**b**) treatment with ML-792, an inhibitor of SUMOlyation; or (**c**) treatment with TNFα/TNFα, IL-1α, and C1q (TIC factors). (**d**) Immunocytochemistry quantification of average vimentin stained iA area in individual iA lines for iA with and without WDR49+ nuclear puncta.

**Figure 4:**
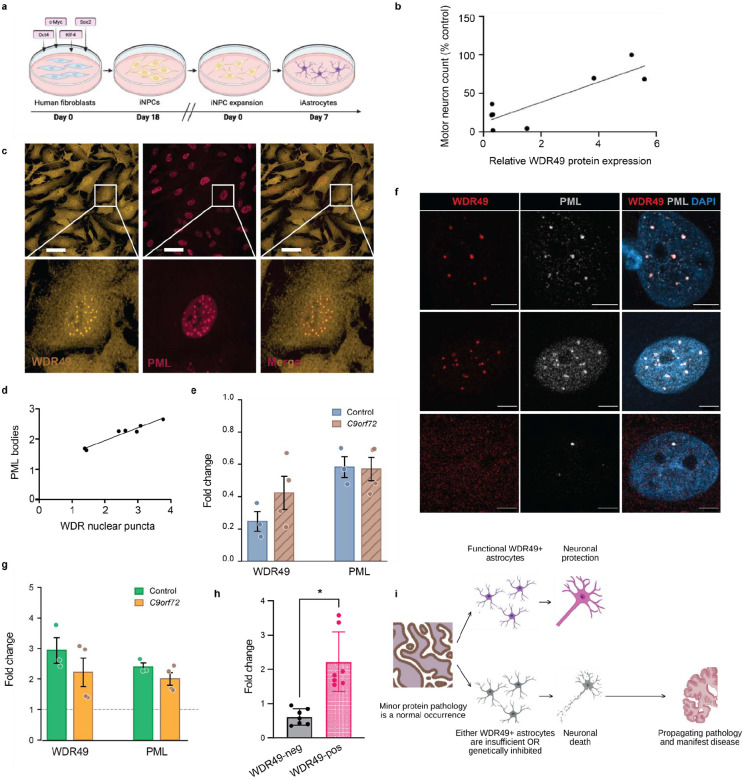
WDR49 expression within iAstrocytes (iA) is associated with neurotoxicity. **(a)** Schematic of the method for developing iA from human fibroblasts.^15^ (**b**) Immunoblotting in iA demonstrates that toxicity to co-cultured primary motor neurons is significantly correlated with WDR49 protein expression. (**c**) Immunocytochemistry for WDR49 and PML in iA derived derived from a neurologically normal control; and (**d**) average counts of PML bodies and WDR49+ nuclear puncta per astrocyte across n=7 iA lines derived from C9orf72-ALS patients and controls; the same lines were used in subsequent analyses. (**e-g**) Immunocytochemistry analysis of change in average counts of PML bodies and WDR49+ nuclear puncta per iA after (**e**) treatment with ML-792, an inhibitor of SUMOlyation; or (**g**) treatment with TNFα. Fold change expressed relative to untreated iA. (**f**) Example immunocytochemistry for WDR49 and PML in untreated iA (upper panels), after treatment with TNFα (middle panels) and after treatment with ML792 (lower panels). (**h**) Change in iA area, a marker of activation, for iA with and without WDR49 nuclear puncta. *p<0.05, ANOVA. (**i**) Model of the role of WDR49 in the development of neurodegeneration.

To determine whether WDR49 plays a functional role in astrocyte neurotoxicity, we used induced astrocytes (iAstrocytes; iA) reprogrammed from ALS patient and control fibroblasts^15^ (Figure 4a), a system which replicates observed disease-specific neurotoxicity of ALS patient postmortem astrocytes.^14,15^ WDR49 protein abundance, measured by immunoblot across iA lines, was a strong positive predictor of co-cultured motor neuron survival (Pearson r^2^ = 0.75, p = 5.7e-3; Figure 4b, Extended Data Figure 4a). This relationship was consistent across genetically heterogeneous donors, suggesting that WDR49 abundance is a determinant of astrocyte mediated neuroprotection.

To understand the cellular machinery through which WDR49 might influence astrocyte function we examined its subcellular localisation. Immunocytochemistry revealed that WDR49 co-localises with PML nuclear bodies in iA (Figure 4c,d). PML bodies are condensates that coordinate SUMOylation-dependent protein modifications and have been linked to EV release.^48,49^ Inhibiting SUMOylation with ML-792 simultaneously depleted PML bodies and WDR49+ nuclear puncta (paired t-test, p < 0.05; Figure 4e, Extended Data Figure 4b). Superresolution microscopy confirmed structural dissolution of WDR49+ puncta upon treatment with ML-792 (Figure 4f). WDR49 is therefore a SUMOylation-dependent constituent of PML nuclear bodies in astrocytes.

We have shown that WDR49+ astrocyte-specific gene expression is enriched for TNF signalling in human tissue, and therefore we tested whether inflammatory stimulation regulates WDR49+ structure and function in iA. Treatment with TNFα, or with the combination of TNFα, IL-1α, and C1q (TIC factors) that induces astrocyte reactivity,^19,50^ increased both PML body number and WDR49+ nuclear puncta number (Figure 4g, Extended Data Figure 4c). Furthermore, change in iA morphology consistent with a reactive response to cytokine stimulation, was strictly contingent on the presence of WDR49+ nuclear bodies (t-test, p = 0.016; Figure 4h, Extended Data Figure 4d). This identifies WDR49 as an upstream regulator of the astrocyte reactivity and a candidate therapeutic target for tuning the astrocyte inflammatory response in ALS.

Together, these *in vivo* and *in vitro* data lead us to propose a model (Figure 4i) in which TDP-43 neuropathology, a feature of normal aging, typically remains subclinical but can progress to propagating neurodegeneration only if it is maintained by a driver, such as a highly penetrant ALS-associated mutation, or if it is unsuccessfully contained by WDR49+ astrocytes. The implication of our model is that failure of WDR49+ astrocytes in isolation is sufficient to cause manifest ALS.

### ALS-associated molecular remodelling within WDR49+ astrocytes

We next characterised the molecular programs distinguishing WDR49+ astrocytes in disease by examining their cell-cell communication landscape, gene regulatory network (GRN) architecture, and enrichment for genetic risk of ALS within their distinguishing gene expression.

**Figure 5.**
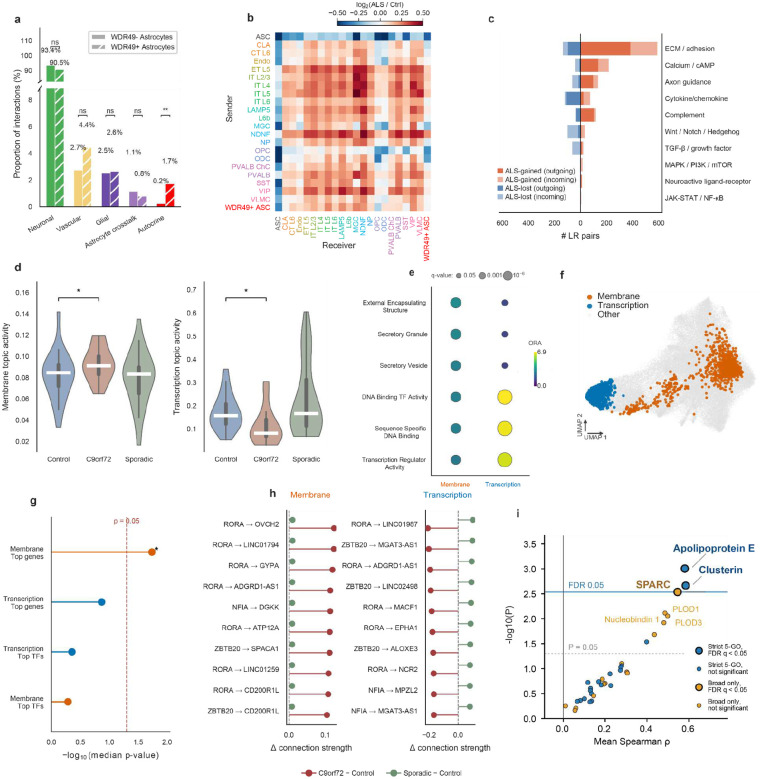
ALS-associated molecular remodelling within WDR49+ astrocytes. (**a**) Proportion of significant ligand–receptor interactions (magnitude rank<0.3, p<0.05) by partner category for WDR49− versus WDR49+ astrocytes. Hatched bars: WDR4+. Per-category Bonferroni, ** p<0.01. (**b**) log_2_(ALS / Control) of significant ligand–receptor pairs (CellPhoneDB, p<0.05) for every sender × receiver pairing across motor cortex cell types. (**c**) KEGG pathway category breakdown of differential ligand–receptor pairs involving WDR49+ astrocytes (|log_2_FC| ≥ 1, status ∈ {gained, lost}). Outgoing pairs (WDR49+ as sender) are shown in saturated color; incoming pairs (WDR49+ as receiver) lightened. (**d**) Violin plots show full distributions across donor metacells for scDORI^54^ topics with different activity in ALS and control tissue. Internal box-and-whisker summarizes the IQR and median (white line). FDR-corrected pairwise contrasts, * q<0.05. (**e**) Curated GO-CC and GO-MF over-representation analysis (ORA) for topics with different activity in ALS and control tissue. Dot color encodes the ORA estimate; dot size encodes q-value. (**f**) UMAP of ATAC peaks embedded by scDORI peak–topic weight vectors. Peaks were selected as the top 1,000 per topic from the peak–topic decoder matrix. Each point is one ATAC peak, coloured by its highest-weight topic: membrane (orange), transcription (blue), or other (grey). (**g**) Genetic enrichment of topic-defining feature sets in ALS rare-variant burden tests.^21^ Lollipops show −log_10_ of the median P-value across genes in each set; the dashed line marks p=0.05. (**h**) Top 10 GRN edges per topic ranked by Δ connection strength (absolute change in TF→target weight × topic-activity shift) in C9ALS − control (red) and sporadic ALS − control (green). (**i**) Volcano plot for astrocyte secreted^57^ proteins with chaperone or protein-stabilising activity. Mean Spearman ρ (x-axis) and smallest −log_10_(p-value) (y-axis) for the correlation between protein level within iA secreted EVs, and measured WDR49 expression per iA line. Blue = proteins annotated to GO:0050821, GO:0061077, GO:0006457, GO:0051082, or GO:1900221; orange = literature-defined (**Supplementary Table 6**). Blue line, FDR = 0.05. Dashed grey horizontal line nominal P = 0.05. Vertical black line at ρ = 0.

Astrocyte reactivity is initiated and maintained through autocrine and paracrine cytokine loops.^51,52^ Applying LIANA+^53^ to quantify ligand-receptor interactions across motor cortex cell types, we found that WDR49+ astrocytes engage a significant number of autocrine signalling interactions relative to WDR49− astrocytes (Figure 5a). This is consistent with our observations that WDR49+ astrocytes both express and respond to proinflammatory cytokines, and could mediate a positive feedback loop enabling astrocyte reactivity. Furthermore, in ALS patients the total set of astrocytes exhibited a net loss of cell-cell communication relative to other cell types (Figure 5b), potentially reflecting disruption of homeostatic glial networks. In contrast, in ALS patients WDR49+ astrocytes gain a set of proinflammatory interactions (Figure 5c).

**Extended Data Figure 5:**
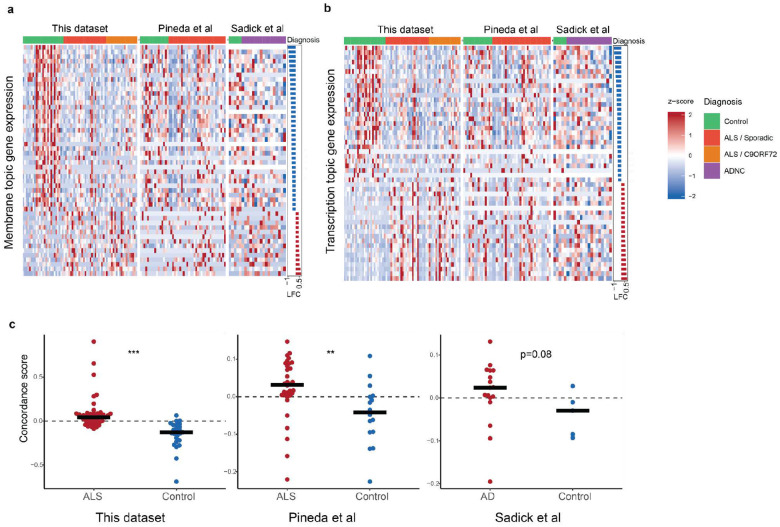
related to ‘Figure 5: ALS-associated molecular remodelling within WDR49+ astrocytes.’ **(a)** Heatmap of top 50 membrane topic genes expression ranked by LFC in our dataset, Pineda et al^3^ and Sadick et al^46^ datasets. Columns are donors, rows are genes. **(b)** Heatmap of top 50 transcription topic genes expression ranked by LFC in our dataset, Pineda et al^3^ and Sadick et al^46^ datasets. Columns are donors, rows are genes. **(c)** Concordance scores for membrane topic across three independent datasets. Each point represents one donor. Score = mean(z-score x sign(Multiome LFC)) across topic genes. Positive values indicate expression aligned with the ALS signature defined in the multiome dataset. *** p<0.001, **p<0.01, Wilcoxon rank-sum test.

To resolve the transcriptional regulatory architecture of WDR49+ astrocytes in ALS patients we applied scDORI^54^ to derive co-regulated topics, which include multiple co-ordinated TFs and their downstream effector genes. Two topics were differentially active between ALS and control WDR49+ astrocytes (Figure 5d). The first was more active in C9ALS patients than controls, and is enriched for genes associated with ‘External Encapsulating Structure’ (GO:0030312; Fisher’s exact test, enrichment = 3.31, p = 4.9e-4; Figure 5e, *“Membrane topic”*), pointing to remodelling of extracellular membrane biology, a process key to extracellular vesicle (EV) release. The other disease-associated topic was less active in C9ALS and enriched with genes associated with transcriptional regulation (Figure 5e). The two disease-associated topics showed significant overlap in their TFs and target genes but diverged significantly in chromatin peak accessibility changes (Figure 5f). This mirrors the pattern of transcriptional regulation we observed across cell-types in ALS motor cortex (Figure 2c). Transcriptional changes associated with both disease-associated topics are discovered in our multi-omic atlas but we see similar changes in an additional single-cell RNA-sequencing study of ALS^3^ and even in prefrontal cortex from Alzheimer’s disease (Extended Data Figure 5a–c).^46^ Of the two disease-associated topics, only the ‘membrane’ topic was enriched with ALS-associated rare genetic variants (Figure 5g), potentially placing loss of function of this gene-set upstream in the pathogenesis of ALS. The most significant ALS-associated genetic variation within the ‘membrane’ topic was within *FLOT2* (Firth logistic regression, OR = 4.4, p = 8.1e-3),^21^ a regulator of EV biogenesis and a canonical marker of EVs.^55^ Regulatory disruption within the ‘membrane’ topic was more pronounced in C9ALS than in sporadic ALS (Figure 5h), suggesting that the *C9orf72* G4C2-repeat expansion exacerbates molecular vulnerability in WDR49+ astrocytes.

### WDR49-dependent astrocyte secretion of protein chaperones

Association of WDR49+ astrocytes with molecular events leading to EV production, and with FLOT2 in particular, points to extracellular vesicle (EV) biogenesis as a disease-relevant pathway. Moreover, this process may be genetically inhibited in ALS patients leading us to hypothesise that EV release from WDR49+ astrocytes is neuroprotective. Our analysis of patient tissue suggested that WDR49 astrocytes may respond directly to the presence of TDP-43 protein aggregates. Propagation of ALS-associated neurotoxicity through the CNS has been linked to extracellular misfolded TDP-43, which can seed pathological aggregates in recipient cells.^56^ We therefore hypothesised that WDR49+ astrocytes may respond to extracellular misfolded protein by secreting EVs containing protein chaperones capable of ameliorating TDP-43 propagation. To evaluate this proposal we tested whether WDR49 expression by iA is correlated with astrocyte secretion of protein chaperones within EVs, which could feasibly ameliorate propagation of TDP-43 pathology.

Astrocyte-secreted protein chaperones were defined as those detected in the mouse astrocyte secretome^57^ and associated with protein misfolding, which produced a list of 34 proteins (**Supplementary Table 6**); we also employed a strict Gene Ontology (GO) driven definition of protein chaperone function leading to a smaller set of 23 proteins. We measured levels of these proteins within EVs harvested from 9 ALS patient-derived iA lines manifesting a range of WDR49 expression. All of the measured proteins were positively correlated with the number of WDR49+ nuclear puncta (Figure 4i) consistent with a model in which WDR49-associated astrocyte activity supports a secretory response to extracellular protein misfolding. Three proteins passed FDR correction: APOE, CLU and SPARC (Spearman rank test, FDR <0.05,Figure 4i, **Supplementary Table 6**). The related protein, SPARCL1, was borderline significant after FDR correction (FDR=0.1, **Supplementary Table 6**).

## Discussion

In this study, we present an integrated single-nucleus multiomic analysis of the ALS motor cortex, combining chromatin accessibility and gene expression profiling across a large cohort of donors. By leveraging both internal and external datasets, we achieve a high-resolution and well-powered characterisation of cell-type-specific molecular alterations in ALS.

At the level of cellular composition, we observe relatively modest changes across major cell classes, with the only significant shift being a reduction in oligodendrocyte proportions in ALS. Notably we demonstrate that *C9orf72* haploinsufficiency, a hallmark of the most common genetic cause of ALS,^47,58^ is specific to oligodendrocytes. Our data suggest that large-scale cell loss or expansion is not the dominant feature of motor cortex pathology. This is consistent with previous works.^59^ Instead, ALS appears to be characterised primarily by functional reprogramming within cell types rather than gross compositional restructuring. We observe coordinated disease-associated changes across cell-types which are driven by a common set of TFs.

Analysis of ALS-associated differential gene expression and chromatin accessibility revealed widespread cell-type-specific molecular alterations, with astrocytes exhibiting the most pronounced changes. The upregulation of chitinases and other reactive astrocyte markers aligns with prior reports of astrocyte activation in neurodegeneration,^29^ reinforcing the notion that astrocytes adopt disease-associated states in ALS. Importantly, the enrichment of ALS genetic risk within astrocyte-specific gene expression signatures provides genetic support for a driver role of astrocytes, rather than a purely reactive response. Despite extensive gene-level analyses, we did not identify individual genes robustly associated with ALS survival after multiple testing correction. However, by shifting to a network-level perspective using PageRank-based gene regulatory network (GRN) inference, we uncover survival-associated regulatory programs.

As an exemplar we characterise WDR49+ astrocytes as a focus of disease-associated transcriptional and regulatory changes, with causal relevance to ALS pathogenesis. WDR49 is specifically expressed in astrocytes in the human CNS.^60^ We find an association between genetic variation within WDR49 and both autosomal dominant^24^ and sporadic ALS.^21^ High impact nonsense mutations are associated with monogenic familial ALS whereas missense mutations are associated with sporadic ALS and lower penetrance. We suggest that the missense variants found in sporadic ALS may only partially reproduce the loss-of-function produced by nonsense variants associated with familial ALS, and thus penetrance may be directly determined by residual WDR49 function. A fully penetrant causal mutation in an astrocyte gene is the first example of ALS underpinned primarily by astrocyte dysfunction.

To define the function of WDR49+ astrocytes and their failure in ALS we studied gene transcription and regulation in this cell type *in vivo*, and the structure and function of WDR49 *in vitro*. Astrocytes toxicity to co-cultured neurons *in vitro* is inversely proportional to their WDR49 expression. *In vitro* and *in vivo* ALS-associated failure of WDR49 is associated with reduced astrocyte reactivity and insufficient EV production, potentially via dysfunction of FLOT2, a membrane protein key to the production of EVs^25,55^ which is also enriched with genetic risk for ALS. WDR49+ astrocytes are enriched in ALS motor cortex and spinal cord tissue and show a spatial association with neuronal TDP-43 pathology. We hypothesise that the neuroprotective effect of WDR49+ astrocytes is mediated by ameliorating the toxicity of misfolded TDP-43 (Figure 3i), which is proposed to be responsible for disease propagation through the CNS.^8,9^ Consistent with this idea, astrocytes secrete EVs containing extracellular protein chaperones in proportion to their WDR49 expression. We note that these protein chaperones include SPARC and SPARCL1 which, in addition to a role in protein stabilisation,^61^ are also involved in stabilisation of synapses^62^ which may be an additional neuroprotective function associated with WDR49+ astrocytes.

Finally we hypothesise that the activity of WDR49+ astrocytes may not be specific to ALS: *WDR49* is co-expressed with *ADAMTSL3*, a gene associated with neuroprotective astrocytes in Alzheimer’s disease.^46^ Moreover, WDR49+ astrocytes up-regulate secretion of EVs containing APOE and clusterin which are well-described in the clearance of β-amyloid pathology in Alzheimer’s disease^63^ and are also genetic drivers of Alzheimer’s disease.^64^

If WDR49+ astrocytes, when functioning normally, are able to ‘prevent’ ALS then they are an attractive therapeutic target. This is particularly true because WDR49 is specifically expressed in astrocytes within the CNS, and even contains astrocyte-specific exons^65^ which could facilitate targeted gene therapy.

## Methods

### 10x Multiome single-nuclei sequencing

#### Sample selection

The multiome study cohort consisted of 70 donors: 25 non-neurological controls, 26 sporadic ALS patients, and 19 patients who suffered ALS associated with G4C2-repeat expansion of C9orf72 (C9ALS). Samples were sourced from four different brain tissue banks (**Supplementary Table 3**). Autopsy tissues were donated with appropriate consent at individual institutions; ethical oversight was provided by the Biomedical Research Alliance of New York (BRANY) IRB except for tissues obtained from the Sheffield Brain Tissue Bank (SBTB) which is approved by Scotland A Research Ethics Committee (Ref. 08/MRE00/103).

#### Sample preparation and sequencing

Nuclei were isolated from approximately 50 mg of frozen tissue using the Nuclei Isolation Kit for Single Cell Multiome ATAC + Gene Expression (10x Genomics) according to the manufacturer’s instructions. Approximately 10,000 nuclei per sample were loaded onto the 10x Genomics Chromium controller using the Chromium Next GEM Single Cell Multiome ATAC + Gene Expression kit and sequenced on an Illumina NovaSeq 6000.

#### Data preprocessing and quality control

Raw .fastq files were aligned to the GRCh38 human reference genome (refdata-cellranger-arc-GRCh38–2020-A) and processed using cellranger-ARC v 2.0 (10x Genomics). Sample QC metrics are provided in **Supplementary Table 1**. Technical artefacts and ambient RNA were removed using CellBender, and doublets were excluded using DoubletFinder. Cell labels were applied via label transfer from the Allen Brain Atlas, and each sample was merged into a single AnnData object, which was used for all downstream analysis. Cells which lacked intersecting RNA and ATAC modalities within the merged object were omitted from downstream analysis, resulting in a final total of 357,862 cells. Separate RNA and ATAC objects were also created, which contained all high-quality cells for their respective modalities, resulting in 585,565 nuclei for the RNA-only object, and 558,921 for the ATAC-only object.

#### snRNA-seq processing

Raw reads were demultiplexed and aligned using Cell Ranger ARC. For each sample, ambient RNA was removed with CellBender. Nuclei were filtered to require ≥ 200 detected genes, genes detected in ≥ 10 nuclei, and < 10% mitochondrial reads. Doublets were identified using Scrublet (score threshold = 0.25, n_prin_comps = 30, top 10% variable genes) and removed. After quality filtering, 586,219 nuclei were retained across 71 samples.

#### snATAC-seq processing

Fragment files from Cell Ranger ARC were processed with SnapATAC2. Nuclei were filtered to require > 1,000 unique fragments and TSS enrichment > 2. Peaks were called using MACS3 on a balanced subset of 1,500 cells per cell type, then merged across samples, yielding 479,421 consensus peaks. After quality filtering, 522,472 nuclei were retained.

#### Cell type annotation

Cell type labels were transferred from a human motor cortex reference atlas (36,449 nuclei).^1^ For RNA, nuclei were normalized to the reference median library size, projected into the reference PCA space (40 PCs), batch-corrected with Harmony, and integrated via Seurat CCA (30 canonical correlates, k_anchor = 5). For ATAC, nuclei were projected into the reference LSI space (40 components), batch-corrected with Harmony, and integrated via Seurat CCA using the same parameters. Cell types were assigned by logistic regression transfer (celltype_lr) with confidence score thresholds of > 0.6 for RNA and > 0.8 for ATAC. This identified 21 cell types; Endo and VLMC were merged as “Vas” (vascular) for differential analyses, and PVALB_ChC was excluded due to small numbers.

#### Integration with external datasets

Our snRNA-seq data were co-embedded with the Pineda et al^3^ ALS motor cortex dataset (74 donors, 265,079 nuclei; disease groups: SALS, C9ALS, pathologically normal) and the Zemke et al. (2023) reference using Seurat CCA integration (40 PCs, 30 CCs), followed by t-SNE (perplexity = 50). RNA-ATAC cross-modality cell type concordance was 86.5% on 359,892 matched nuclei.

#### Genetic enrichment for ALS risk within cell-specific gene expression

Rare-variant burden statistics based on the “singleton high-impact variants” model were used for scDRS analysis. Following the recommended procedure, the top 1,000 ranked genes based on their p-values were retained. Both the reference dataset and our own dataset (excluding disease samples) were used as comparison. Internal scDRS filtering was enabled, and 1,000 matched control gene sets were used to estimate empirical significance. For each cell, scDRS returned normalized disease scores, Monte Carlo p-values, and z-scores for downstream analysis. To identify ALS-enriched cell types, cells with scDRS Monte Carlo p-value < 0.05 were defined as significant cells. For each cell type, a one-sided Fisher’s exact test was performed to evaluate whether significant cells were overrepresented in that cell type relative to all remaining cells. The resulting Fisher p-values were further adjusted using the Benjamini–Hochberg procedure.

#### Pseudobulk differential analysis

Pseudobulk expression and accessibility profiles were generated by summing counts across nuclei within each donor × cell type combination, requiring > 100,000 total RNA counts or > 500,000 ATAC fragments. Two donors with unknown sex were imputed as male based on Y-chromosome gene expression. Differential analysis used PyDESeq2 with design ~Type + Sex. Pairwise contrasts (C9ORF72 vs. Control, Sporadic vs. Control) were computed per cell type. Genes and peaks on chrX/Y were excluded to avoid residual sex confounding (2,847 genes, 9,847 peaks). For gene analysis, genes with total counts < 10 were excluded. For peak analysis, peaks detected in < 5% of nuclei in both groups were excluded; no fold-change pre-filter was applied. Significance thresholds: adj. P < 0.05 and |log2FC| > 0.25.

#### DEG classification by cell-type specificity

The top 200 genes by |Wald statistic| per cell type × disease comparison were unioned (4,081 genes), then classified: Category 1 required ≥ 90% sign consistency across all 38 cell type × disease columns with mean |stat| ≥ 1.5; Category 2 required the same within neurons only; Category 3 required max non-neuronal |stat| ≥ 3 with mean neuronal |stat| < 1.5.

#### Gene ontology enrichment

Hypergeometric tests were performed against GO Biological Process 2023 (Enrichr). For each signed subcategory, the background comprised genes expressed at mean CPM ≥ 1 in the relevant cell types (~21,000–23,000 genes). P-values were BH-corrected. Terms were validated for robustness at a CPM ≥ 10 background; categories with no surviving terms (Cat. 1 DOWN, Cat. 3 DOWN) were excluded.

#### Motif enrichment

Top 1,000 DARs per direction per cell type were analyzed with HOMER findMotifsGenome.pl (window = 500 bp, known motifs only, disease-direction peaks vs. opposite-direction background). A signed enrichment score (−log10 P in disease-UP minus −log10 P in disease-DOWN) was computed per TF × cell type × disease. TFs with mean |score| ≥ 5 in non-neuronal cells (53 TFs) were collapsed into 16 groups by Pearson r > 0.85 clustering and biological family annotation.

#### Gene regulatory network construction and PageRank

Pseudobulk RNA and ATAC profiles used for GRN construction were normalized to 10,000 counts per pseudobulk and log1p-transformed. Donor × cell-type pseudobulks with fewer than 15 contributing nuclei were excluded. Within each cell type, genes and peaks below the 25th percentile of donor-wise variance were filtered out, and genes were further restricted to those with mean pseudobulk expression > 0.001 and detection in >15% of donors.

Cell-type-specific GRNs were assembled as directed TF → target-gene networks by joining three mappings (TF → motif → peak → gene). TF–motif relationships were taken from JASPAR and CIS-BP; motif occurrences in the consensus peak set were identified per cell type with FIMO (MEME Suite); peak–gene links were defined for any peak within ±100 kb of a gene’s TSS using GENCODE v30 coordinates. For each cell type, Spearman correlations across donors were computed for every TF–gene and every peak–gene pair. For every motif occurrence linking a TF to a candidate target through a peak, an edge score was defined as the geometric mean of the absolute TF–gene and peak–gene correlations, Peak_Score = √(|ρ_{TF,gene}| · |ρ_{peak,gene}|). Multiple motif/peak instances supporting the same (TF, gene) pair were collapsed by taking the maximum, producing an Importance weight per edge. Edges below the 90th percentile of Importance per cell type were discarded, yielding the final regulatory network used for PageRank.

For PageRank propagation, the directed adjacency A was transposed so that flow runs from regulator to target, self-loops were removed, sink nodes (zero out-degree) were assigned a self-loop to absorb dangling mass, and rows were normalized to unit sum to obtain a transition matrix P. Personalized PageRank was computed on P with damping α = 0.85; the personalization vector w varied by analysis as described below.

For disease-associated PageRank, gene-level personalization weights were set to the unsigned per-gene effect size (AUROC − 0.5) from the Sporadic vs. Control, C9ORF72 vs. Control, and ALS vs. Control contrasts, yielding a per-cell-type weight matrix W (contrasts × genes). Significance of each gene’s centrality was assessed against a null generated by independently shuffling the elements of each row of W (1,000 permutations per cell type), with per-gene z-scores computed as z = (π_obs − μ_null) / σ_null. For donor-personalized PageRank, the personalization vector for each donor in a given cell type was set to that donor’s pseudobulk RNA profile normalized as above (CPM10k + log1p); the resulting scores were used as donor-level features for downstream analyses (e.g., survival analysis).

#### Survival analysis

Cox proportional hazards regression tested donor-level PageRank TF scores against survival (months from onset to death) in 33 ALS patients (17 C9ORF72, 16 Sporadic), adjusting for age of onset and sex. Kaplan-Meier curves stratified patients by median TF score.

#### Cell type proportion analysis

Per-donor proportions from RNA assignments were compared between disease groups using Wilcoxon rank-sum tests (BH-corrected). The analysis was performed independently in our cohort and.^3^

#### STREAM trajectory analysis

STREAM (Single-cell Trajectories Reconstruction, Exploration and Mapping of single-cell data; Chen *et al*., 2019) was employed to determine if WDR49+ astrocytes formed part of an astrocytic developmental trajectory. Briefly, STREAM constructs elastic principle graphs which capture the global topology of single-cell trajectories and enable fine-tuning of graph complexity to enhance trajectory inference. STREAM also generates trajectories agnostic of cell metadata, making it suited to assessing disease specific cell-states in an unbiased manner. Astrocytes from our dataset were subsetted from the previously constructed integrated RNA object, leaving a total of 82,005 astrocytes. STREAM was then applied to construct an astrocyte specific trajectory. Astrocytes were filtered based on gene expression (min_n_features = 100) and genes were filtered based on global expression (min_n_cells = 5). Gene expression was normalised based on library size and log transformation was applied. Dimensionality reduction was then applied using top principal components, and trajectory inference was performed using 10 clusters to seed the elastic principal graph, followed by learning using epg_alpha = 0.01, epg_mu = 0.05 and epg_lambda = 0.01. Trivial branches were pruned (epg_collapse_par = 2), and graph growth was controlled with incr_n_nodes = 30 and epg_ext_par = 0.8. Following graph construction, gene expression of WDR49 was projected on a flat tree visualisation of the principal graph to identify WDR49-enriched trajectory branches.

#### Cell composition analysis

To determine ALS-associated changes in cell type proportions, we applied scCODA, a Bayesian compositional analysis framework, implemented via the pertpy^66^ Python package. The false discovery rate (FDR) was set to 0.2, consistent with the discovery threshold applied to all real-data analyses in the original scCODA publication.^27^ Single-nucleus observations were aggregated at the donor level. Seven pre-specified contrasts were evaluated, namely (i) ALS (*C9orf72*-ALS and sporadic ALS donors combined) versus non-neurological controls; (ii) *C9orf72*-ALS versus controls; (iii) sporadic ALS versus controls; (iv) C9orf72-ALS versus sporadic ALS; (v) C9orf72-ALS versus sporadic ALS with post-diagnosis survival duration included as a continuous covariate; (vi) ALS donors only, with survival duration as the sole covariate; and (vii) all donors, with age at death as a continuous covariate. For each contrast, cells were subset to the relevant donor groups prior to MuData construction; donors with missing covariate values were excluded from contrasts in which that covariate appeared. For contrasts involving survival or age at death, each covariate was verified to take a single value per donor before model fitting.

Model inference was performed using the No-U-Turn Sampler (NUTS) on CPU via JAX. Credible compositional changes were identified by calling the function credible_effects at the pre-specified FDR of 0.2; the corresponding posterior inclusion probabilities and log-scale effect sizes (beta) are reported for each contrast. For each contrast, per-donor compositional matrices, full posterior summary statistics (mean effect, 95% highest density interval), and credible effect tables were saved.

#### scDORI framework

scDORI (Single-cell Deep Multi-Omic Regulatory Inference)^54^ was used to capture the full landscape of gene regulatory networks (GRNs) within WDR49+ astrocytes. scDORI is a scalable computational framework that jointly models paired RNA-seq and ATAC-seq data to infer continuous, enhancer-mediated gene regulatory networks (eGRNs) at single-cell resolution. scDORI employs an encoder–decoder architecture in which mechanistically constrained decoders enforce established regulatory logic (including TF chromatin binding, enhancer-gene links, and TF-target gene co-expression) to decompose the data profile of each cell into a mixture of latent regulatory modules termed “Topics”. Each Topic represents a set of TF-target gene relationships mediated by specific cis-regulatory elements, enabling continuous, cell-specific resolution of GRN activity without relying on predefined cell-type annotations.

We applied scDORI to the astrocyte-only subset of our multiome dataset. Nuclei annotated as “WDR49+ Astrocytes” (as per the STREAM analysis) or “Other Astrocytes” were extracted from the full RNA and ATAC AnnData objects, retaining both modalities. Cells were intersected, and mitochondrial genes were removed before downstream processing.

##### Phase 1: Preprocessing.

All preprocessing was performed using the scDORI preprocessing pipeline (scdori.pp) against the human reference genome (hg38). ATAC peak coordinates were parsed from the variant index format (chr-start-end). Reference genome files (annotation GTF and chromosome sizes) were downloaded for hg38 and used throughout. Four thousand highly variable genes (HVGs) and transcription factors (TFs) were selected using a TF list derived from the CisBP human motif database (cisbp_human.meme). The TF list was padded to exactly 300 entries by supplementing with the most variably expressed genes present in both the motif database and GTF annotation. Metacells were constructed using Leiden clustering at resolution 5.0, with donor identity as the batch correction key. Peak selection retained all promoter-proximal peaks and the most highly variable distal peaks, yielding a final set of 90,000 ATAC peaks per metacell. ATAC metacells were subsequently subsetted to this filtered peak set.

Transcription factor binding affinity was estimated by computing motif scores across all selected peaks using the CisBP position weight matrices. In-silico ChIP-seq activator and repressor matrices were then derived by integrating per-metacell TF expression (from the RNA modality) with peak accessibility and motif scores. A gene-peak distance matrix was constructed by computing the distance between each gene and each ATAC peak within an 80 kb window. Raw distances were transformed using exponential decay with a scaling factor of 20,000 bp (d_transformed = exp(-d / 20,000)), and values below the minimum cutoff (0.0183) were set to zero, preserving only biologically proximal gene-peak pairs.

The scDORI model was initialised with 20 latent topics and a two-layer encoder architecture (256 dimensions per layer). Priors were seeded from the gene-peak distance matrix and the *in-silico* ChIP-seq activator and repressor matrices using warmup-phase parameter initialisation. Donor identity was encoded as a one-hot batch covariate (one level per donor) to account for inter-donor technical variation.

Metacells were split into training (80%) and evaluation (20%) sets using a fixed random seed (seed=42). The model was trained using train_scdori_phases with the following hyperparameters: learning rate 5×10^−4^; L2 regularisation on topic-TF weights (λ=10^−3^) and topic-peak weights (λ=10^−4^); L1 regularisation on topic-TF weights (λ=10^−4^). All other L1/L2 terms were set to zero. Training was executed on an NVIDIA Tesla P100 GPU (128 GB RAM, 16 CPUs; Stanford SCG cluster), with a maximum wall time of 30 hours. Best Phase 1 weights were saved and used to initialise Phase 2.

##### Phase 2: GRN training.

Phase 2 refined the per-topic gene regulatory networks, loading Phase 1 weights and reinitialising with GRN-phase parameter settings. All model architecture and regularisation hyperparameters were identical to Phase 1. The per-cell batch size was reduced to 16 to accommodate the expanded peak space (~90,000 peaks) within GPU memory limits, and gradient checkpointing was applied to the forward pass to further reduce peak memory allocation. Training was executed on an NVIDIA A100 (80 GB) GPU (128 GB RAM, 16 CPUs; Stanford SCG cluster), with a maximum wall time of 168 hours. Final GRN weights were saved for downstream inference.

Topic activations were extracted from the trained scDORI latent space across all astrocyte metacells. Topics were ranked by mean activation in WDR49+ astrocytes, and the ones with mean activation above 0.07 were retained as active. Topic gene programmes were characterised by over-representation analysis (ORA) of Gene Ontology Cellular Component terms using the MSigDB resource via decoupler-py. Genetic enrichment within topic gene programmes was assessed by Wilcox rank sum test.

Differential GRN edge activation was quantified by weighting the combined activator and repressor GRN edge strength (|GRN_act| + |GRN_rep|) by the difference in mean topic activation between each ALS subtype (C9orf72 ALS, Sporadic ALS) and controls, yielding a difference in edge weight (Δ) per TF–target pair. The top 10 edges by maximum absolute Δ across subtypes were reported per topic. TF importance within each topic was summarised as a within-topic z-score derived from scDORI activator and repressor GRN matrices, and the top five globally ranked activator and repressor TFs were visualised. Differential topic activation across disease groups in WDR49+ astrocytes was tested using weighted least squares regression on centred log-ratio (CLR) transformed donor-level pseudobulk topic profiles, with FDR correction applied across topics.

#### Cell-cell communication analysis with LIANA+

In order to characterise ligand–receptor-mediated intercellular signalling within ALS and non-neurological control motor cortex, we applied LIANA+,^53^ a scalable framework that integrates multiple ligand–receptor inference methods around a common knowledge base. Analyses were performed independently in ALS donors (C9orf72-ALS and sporadic ALS combined) and non-neurological controls. For each condition, single-nucleus transcriptomic observations were aggregated by cell type. Ligand–receptor interactions were scored using the rank_aggregate consensus method, which aggregates interaction evidence across the full suite of methods in LIANA+ (CellPhoneDB, CellChat, NATMI, Connectome, SingleCellSignalR and scSeqComm), producing a unified rank-based score that jointly captures interaction magnitude and cell-type specificity. Ligand–receptor pairs were drawn from the LIANA+ consensus resource, and statistical significance was assessed by permutation testing using 1,000 permutations of cell-type labels, providing empirical p-values for each ligand–receptor pair per directed cell-type pair. All interaction results were retained for downstream comparison between conditions.

### Genetic association testing

Enrichment of genetic variation in WDR49 in sporadic ALS cases compared to controls was performed using 13,138 ALS patients and 69,775 controls as described previously^21^. Variants were included at MAF < 0.001 which alter the amino acid sequence. Statistical enrichment was calculated using SKAT^42^ with sex, the first 10 principal components (PCs) of the genotype matrix, and the number of synonymous variants per individual as covariates.

### Neuropathology

Tissue blocks (n=16 blocks from n=10 cases) with patchy WDR49 astrocytosis were investigated to assess the relationship between WDR49 astrocytosis and TDP43 proteinopathy. Adjacent sections were immunostained for phosphorylated TDP43 and WDR49 and digitised using a Hamamatsu Nanozoomer slide scanner. The WDR49- and phosphoTDP43-stained sections were aligned and viewed simultaneously. Four circular ROIs of 1mm radius were highlighted on the WDR49-stained slide, blind to the pTDP43 status by a qualified neuropathologist (JRH). The corresponding areas were then highlighted on the pTDP43 stained slide. These ROIs were: an area of white matter that was positive for WDR49 astrocytosis with an area of overlying cortex adjacent to this and an area of white matter that was negative for WDR49 astrocytosis with an area of overlying cortex adjacent to this. pTDP43 inclusions were then counted in glia in both the white matter and the cortex, and in neurons in the cortex by a scientist trained in MND tissue studies (FYM). For each type of tissue (white matter vs Cortex) and cell (neuron vs glia) regions with and without WDR49 astrocytosis were compared for the number of pTDP43+ inclusions by Wilcoxon matched-pairs signed-ranks test. All showed significantly more TDP43 proteinopathy in regions with WDR astrocytosis (white matter glia p=0.049; cortical neuron pathology p=0.015 and cortical glia pathology p=0.02).

Sections of the cervical and lumbar spinal cord were stained for WDR49 in 5 controls and 9 cases of sMND-TDP. Similar to the pattern seen in the precentral gyrus, There was generally more expression in the grey matter than the white matter. Expression was largely granular and variously seen in the vicinity of blood vessels (possibly representing end-/foot-processes of astrocytes), around astrocytes away from blood vessels and isolated puncta in the neuropil.

In the corticospinal tracts of 6 out of 9 sMND-TDP cases and no controls, there was WDR49-astrocytesis, similar to that seen in the precentral gyrus. Again, there was variable expression, which was present at both levels in four cases and only at one level (cervical) for two cases.

In the grey matter of the cord, neurons were largely negative in cytoplasm and nucleus, with the exception of the nucleolus. This contrasts to the motor cortex, where there is more neuronal cytoplasmic labelling.

### Induced astrocyte (iA) in vitro modelling

#### Differentiation of iA

Skin fibroblasts were directly converted to iNPCs using the protocol described in Meyer et al.^15^ Fibroblast samples were obtained via the University of Sheffield ALS biosampling programme (Study number STH16573, Research Committee Reference 12/YH/0330). Informed consent was obtained from all donors prior to biopsy collection. Directly induced NPCs were cultured on fibronectin-coated plates in DMEM/F12 with glutamax, N2 supplement, B27 supplement and bFGF (40 ng/mL). Differentiation to astrocytes was initiated by changing the media to DMEM, FBS (10%), N2 (0.2%) and 1% penicillin streptomycin for 7 days.^67^ iAstrocytes were passaged using Accutase for 5 minutes, with a 4-minute centrifugation at 200G before seeding at a density of 20,000 cells/cm2. Cells were treated with TNFa (300–01A) at a concentration of 25ng/ml for 48 hours.

#### Immunocytochemistry

iAstrocytes were fixed in 4% PFA for ten minutes. Cells were permeabilised in 0.3% Triton X-100 for ten minutes before blocking in 5% donkey serum for 1 hour. Primary antibodies were added to the cells overnight at 4oC (Vimentin (ab5733), 1:1000; WDR49 (HPA036226), 1:300; PML (ab96051), 1:500) before washing thrice with PBS. Secondary antibodies were added for 1 hour at room temperature (donkey anti-chicken (A78948), donkey anti-mouse (A31571), donkey anti-rabbit (A10042), all 1:1000). Hoescht was added to the cells for ten minutes at a concentration of 1:6000 before cells were washed three times. Cells were imaged using the Opera Phenix high-throughput scanning confocal microscope (Revvity). Analysis was performed using Harmony software (Revvity).

#### Small extracellular vesicle (sEV) isolation and concentration

For EV-conditioned medium collection, day 6 iAstrocyte cultures were washed twice with PBS and switched to FBS-free medium consisting of DMEM supplemented with 10% knockout serum replacement, 0.2% N2 supplement and 1% Penicillin-Streptomycin. After 24 h, conditioned medium was collected, centrifuged at 300 × g for 5 min at room temperature to remove debris, and the supernatant was stored at −80°C until further processing.

Cell culture media was first clarified by centrifugation at 2,000 ×g for 20 minutes at 4 °C. The supernatant was then filtered through a 0.22 μm vacuum filter system (Nalgene). The filtrate was concentrated using a 10 kDa molecular weight cut-off (MWCO) centrifugal concentrator (Vivaspin). sEVs were isolated from 0.5 mL of concentrated media by size-exclusion chromatography (SEC) using a qEVoriginal/35 nm column (Izon Science) coupled to an automatic fraction collector. The column was equilibrated and eluted with phosphate-buffered saline (PBS, Gibco). The void volume was set for 2mL and particle-rich eluate collected was 2.5 mL. The collected EVs were further concentrated using a 10 kDa MWCO centrifugal concentrator (Vivaspin).

#### Nanoparticle Tracking Analysis (NTA)

Isolated particle size distribution and concentration were analysed by Nanoparticle Tracking Analysis (NTA) using a NanoSight NS3000 instrument (Malvern Panalytical). Samples were injected at a constant flow using a syringe pump with the flow rate set to 50 (arbitrary units). Video captures were recorded for 90 seconds per sample with the camera level set to 14. All NTA measurements were performed on the day of isolation. The mean diameter of isolated vesicles was <200 nm and the mode diameter was <150 nm, indicating enrichment of sEVs.

#### EV lysis

Isolated EVs were lysed in RIPA buffer (Sigma-Aldrich) supplemented with a protease inhibitor cocktail (Sigma-Aldrich, P9599). The samples were incubated on ice for 30 minutes to facilitate lysis, with intermittent sonication in an ice-cold water bath. Sonication was performed for 1 minute at 5-minute intervals. The total protein concentration of the lysates was subsequently determined using a micro bicinchoninic acid (microBCA) assay kit (Thermo Fisher Scientific), according to the manufacturer’s instructions.

#### Mass spectrometry analysis

12.5 μl of each EV sample was added to the same volume of 2X S-trap lysis buffer to achieve a final concentration of 5% SDS/50 mM Tris, pH 7.4. Samples were reduced with TCEP to a final concentration of 5 mM and heated at 70 °C for 15 minutes. Proteins were alkylated by adding Iodoacetamide (IAA) to a final concentration of 10 mM, and the samples were incubated at 37 °C in the dark for 30 minutes. 2.5 μl of 27.5 % phosphoric acid and 165 μl of S-Trap binding buffer (90% methanol, 100 mM TEAB, pH 7.1) were then added to each sample. Samples were then loaded into S-Trap columns (ProtiFi) by centrifugation at 10,000 x g for 60 seconds. Samples were washed three times with 225 μl of S-Trap binding buffer through centrifugation at 10,000 x g for 60 seconds. 1 μg of Trypsin (Pierce, sequencing grade) was added to each sample in a total digestion volume of 20 μl of 50 mM TEAB, and digestion was performed at 47 °C for 1 hour, followed by 37 °C for 1 hour. Peptides were eluted with the addition of 40 μl of 50 mM TEAB, 40 μl of 0.2% aqueous formic acid, and then 40 μl of 50% acetonitrile with 0.2% formic acid, followed by centrifugation at 10,000 x g for 60 seconds for each elution. Pooled eluted peptides were dried in a vacuum concentrator and resuspended in 0.5% formic acid for LC-MS/MS analysis. Each sample was analysed using nanoflow LC-MS/MS using an Orbitrap Exploris 480 (Thermo Fisher) mass spectrometer equipped with an EASY-Spray source coupled to a Vanquish LC System (Thermo Fisher). Peptides were desalted online using a Pepmap Neo C18 nano trap column, 300 μm I.D. × 5 mm (Thermo Fisher) and then separated using an EASY-Spray column, 50 cm × 75 μm ID, PepMap Neo C18, 2 μm particles, 10 Å pore size (Thermo Fisher) and separated using a 60-minute gradient. The Orbitrap Exploris 480 was operated in positive mode using a Velocity DIA method. MS1 spectra were acquired at a resolution of 60,000 at m/z 200 with a normalised AGC target of 300%. For the DIA analysis, multiply charged precursors in 40 m/z bins (400–900 m/z) were accumulated with a normalised AGC target of 800%, and subjected to HCD fragmentation (145–1500 m/z) with a collision energy of 30%.

Raw mass spectrometry data files were processed with DIA-NN version 2.3.0. Data were analysed in library-free mode using a peptide library predicted from a Human proteome Fasta file (downloaded Aug 2025) containing 20475 proteins. The following settings were used in library prediction. Trypsin was set as the protease, with a maximum of 1 missed cleavage. Cysteine carbamidomethylation was enabled as a fixed modification. The maximum number of variable modifications was set to 1, and protein N-term Met excision was set as a variable modification. An FDR of 1% used for identification-level cut-offs. The DIA-NN output was loaded into Perseus version 1.6.10.50, and the matrix was filtered to remove all proteins that were potential contaminants and decoy hits. LFQ intensities were log2(x)- transformed, and data were filtered to retain proteins with a minimum of two peptides and at least 3 valid LFQ intensities in one group. Data were normalised by subtracting column medians, and missing values were imputed from the normal distribution with a width of 0.3 and downshift of 1.8.

#### Definition of astrocyte secreted protein chaperones

To determine whether WDR49 expression within iA was associated with abundance of astrocyte-secreted proteins linked to processing of misfolded proteins we defined a set of 34 proteins which are detected in the mouse astrocyte secretome^57^ and associated with protein misfolding (**Supplementary Table 6**). We also employed a strict Gene Ontology (GO) driven definition of 23 proteins which are present in the astrocyte secretome as defined by^57^ and within one or more of the following GO terms: “protein stabilization” (GO:0050821), “chaperone-mediated protein folding” (GO:0061077), “protein folding” (GO:0006457), “unfolded protein folding” (GO:0051082), and “β-amyloid clearance” (GO:1900221). Correlations with measures of WDR49 expression were determined by a Spearman rank test and FDR corrected for multiple testing.

## Extended Data

## Supplementary Material

This is a list of supplementary files associated with this preprint. Click to download.

- SupplementaryTablesupdated.xlsx

**Supplementary Table 1:** Metadata for ALS patient and control primary motor cortex samples used to generate single-nucleus transcriptomes and chromatin accessibility profiles.

**Supplementary Table 2:** Cell proportions per sample within primary motor cortex.

**Supplementary Table 3:** ALS-associated gene expression changes per cell type within primary motor cortex.

**Supplementary Table 4:** ALS patient survival-associated gene expression changes per cell type within primary motor cortex by Cox regression.

**Supplementary Table 5:** Gene Ontology (GO) categories enriched within genes upregulated across all cell-types.

**Supplementary Table 6:** Astrocyte secreted protein chaperones correlated with WDR49 iA phenotypes.

## Data Availability

All raw RNA-seq data can be accessed via the National Center for Biotechnology Information’s Gene Expression Omnibus database (Bioproject ID is PRJNA1468995). In addition, we provide an interactive R Shiny app to visualize the gene expression and other clinical variable associations (https://sbonsall.shinyapps.io/ALS_Genetics_Browser/). Code is deposited at github repository: https://github.com/zhoujt1994/ALS_Multiome.git
